# Adherence to Hypothermia Guidelines: A French Multicenter Study of Fullterm Neonates

**DOI:** 10.1371/journal.pone.0083742

**Published:** 2013-12-31

**Authors:** Marie Chevallier, Anne Ego, Christine Cans, Thierry Debillon, on behalf of the French Society of Neonatology

**Affiliations:** 1 Neonatology and Pediatric Intensive Care Unit, Grenoble University Hospital, Grenoble, France; 2 Clinical Research Center (CICO3), Grenoble University Hospital, Grenoble, France; 3 THEMAS (Techniques pour l'évaluation et la modélisation des actions de santé), Joseph Fourier University-Grenoble1, Grenoble, France; Hôpital Robert Debré, France

## Abstract

**Aim:**

The objective of this study was to describe the French practice of hypothermia treatment (HT) in full-term newborns with hypoxic-ischemic encephalopathy (HIE) and to analyze the deviations from the guidelines of the French Society of Neonatology.

**Materials and Methods:**

From May 2010 to March 2012 we recorded all cases of HIE treated by HT in a French national database. The population was divided into three groups, "optimal HT" (OHT), “late HT” (LHT) and “non-indicated” HT (NIHT), according to the guidelines.

**Results:**

Of the 311 newborns registered in the database and having HT, 65% were classified in the OHT group, 22% and 13% in the LHT and NIHT groups respectively. The severity of asphyxia and HIE were comparable between newborns with OHT and LHT, apart from EEG. HT was initiated at a mean time of 12 hours of life in the LHT group. An acute obstetrical event was more likely to be identified among newborns with LHT (46%), compared to OHT (34%) and NIHT (22%). There was a gradation in the rate of complications from the NIHT group (29%) to the LHT (38%) group and the OHT group (52%). Despite an insignificant difference in the rates of death or abnormal neurological examination at discharge, nearly 60% of newborns in the OHT group had an MRI showing abnormalities, compared to 44% and 49% in the LHT and NIHT groups respectively.

**Conclusion:**

The conduct of the HT for HIE newborns is not consistent with French guidelines for 35% of newborns, 22% being explained by an excessive delay in the start of HT, 13% by the lack of adherence to the clinical indications. This first report illustrates the difficulties in implementing guidelines for HT and should argue for an optimization of perinatal care for HIE.

## Introduction

The incidence of hypoxic ischemic encephalopathy (HIE) in newborns is currently imprecise with numbers ranging from 1 to 8 per 1000 live births worldwide [1], [2]. The method of identifying cases, the definition of HIE and the source study population have an impact on the reported incidence [3], [4]. In France, apart from a study by Pierrat *et al* in the Nord Pas de Calais region, where an incidence of 0.86 per 1000 was found, little precise epidemiological data on HIE is available [5]. However the prognosis is severe and mortality can reach 20 to 40% of cases. The rate of adverse outcomes (death, cerebral palsy, severe cognitive deficit) reaches 30 to 50% in cases with Sarnat stage II HIE and 100% for stage III [6]–[8]. Some studies report a 5–6 increased risk of epilepsy [10], [11].

Over the last fifteen years, several randomized trials on the neuroprotective effect of hypothermia (HT) have been published [12]–[18]. The results are summarized in four meta-analyzes, which show a reduction of 25% in the combined risk of death and major neuro-developmental disability at 18 months (RR  =  0.76 [0.65 to 0.89]) [18]–[21]. Currently, HT is the standard treatment for HIE, and in 2009, the French Society of Neonatology (SFN) published guidelines on both the indications for HT and how it should be performed [22]. The role of HT on the medium and long term prognosis of these children remains unclear. Databases set up in England and the USA (Toby Cooling Register and Vermont Oxford Register) could help provide answers to this question [9], [23], [24]. In France, in May 2010, the SFN set up a database with similar objectives. It is intended to register all HIE cases admitted to neonatal intensive care units (NICU), whether treated or not with HT, and to ensure their subsequent follow-up.

To our knowledge, few studies have been published on the evaluation of the practice of HT in the various countries where this treatment is recommended. This is necessary before any assessment of the long term impact of this new treatment can be made. The aim of this study was to describe the practice in France for full-term newborns with HIE treated by HT, and to analyze deviations from the SFN guidelines and the reasons for these.

## Materials and Methods

Since May 2010, the full-term neonate HIE database records newborns of gestational age (GA) ≥ 36 weeks, weighing ≥ 1800g and presenting with HIE (mild, moderate or severe), regardless of the treatment (HT or standard care). All French Level III NICU (including overseas territories) have secure access and reporting of cases is declarative, without control of completeness. During the study period, 33 level III NICUs among 57 nationwide included newborns in the database, with a mean number of infants reported per center of 9.2 (+ / –8.2). As of March 22, 2012, the database contained 465 cases registered in 23 months. Not all French Level III NICU participated, so the number of newborns included is not exhaustive.

### Ethics statement

Data collection was approved by the French authority entitled “*Commission Nationale de l’Informatique et des Libertés” (*National Data Protection Authority, Ref: AT/FLR/DI103637, Authorization N°1426721, 2010–266). This data collection was initiated in 2010 to study the newborns with HIE and to assess the implementation of the hypothermia treatment in France. No written consent from the parents was required by this French authority. According to their guidelines for an observational study, we advised clinicians to provide a parent information leaflet about data collection. If parents express a disagreement with this survey, none data collection on their child was performed but we did not collect this information from each centers. The main finality of this data base, specified by the approving of the *Commission Nationale de l’Informatique et des Libertés,* is to use the data for statistical and epidemiological analysis on order to ameliorate the management of newborns with HIE. For that, we use anonymous data.

### Selection of the study population and variables studied

**Figure 1:**
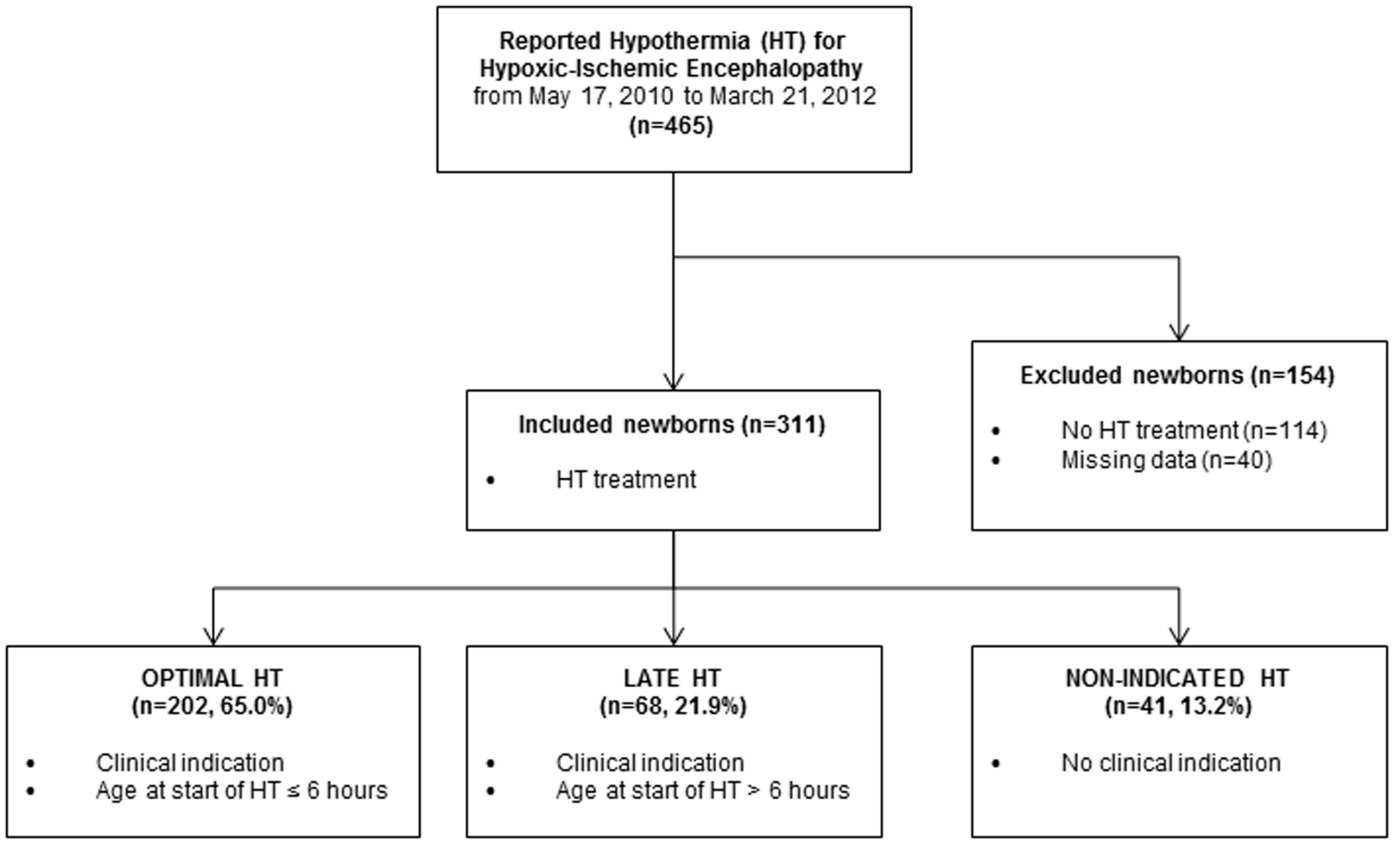
Flow chart of the population study.

Our study included all newborns reported in the database between May 2010 and March 2012 except newborns with missing data concerning at least one of the criteria for the indication of HT (n = 40) and those with HIE but not treated by HT (n = 114) (Figure 1). According to our definition, a lack of clinical indications was observed for 58 newborns (50.9%) among 114. Among the remaining 56 untreated cases, the main reasons reported by neonatologists were the lack of electrophysiological anomalies or clinical indications for HT (n = 16), the presence of contraindications (n = 15), late admission to the NICU (n = 13) and the restriction of neonatal care for critically ill neonates (n = 11).

The variables recorded in the database and analyzed were:

1. Delivery characteristics (place, date, inborn or outborn, time to admission),
2. Clinical and paraclinical characteristics of the neonate (term, weight, sex, care in the delivery room, clinical stage of HIE severity, temperature on admission, umbical cord acid-base balance (or within the first hour of life), EEG performed before initiation of HT,
3. Conditions of HT (time, material used),
4. Short-term outcomes (death, clinical examination results according to the Amiel-Tison scoring [25] and brain imaging data at discharge). For the neurological examination, 3 degrees of neurological abnormalities were retained (normal or mild, moderate, and severe). For cerebral imaging, 4 types of abnormalities were distinguished i) basal ganglia and/or cortical or subcortical lesions, ii) white matter lesions apart from hemorrhagic petechial lesions, iii) isolated hemorrhagic petechial lesions, iii) brainstem or cerebellar lesions.
5. Adverse events associated with HT such as thrombocytopenia and bleeding disorders, hemorrhage, inadvertent overcooling, overheating, hypomagnesemia, and skin lesions.
6. Visceral complications linked to hypoxia-ischemia (HI) such as liver cytolysis or failure, shock or isolated hypotension, hypertension, renal failure, pulmonary hypertension, glucose intolerance and a paralytic ileus.

### Definition of optimal and sub-optimal HT

According to the SFN guidelines, the clinical, paraclinical, and organizational criteria justifying the initiation of HT are the following:

1. GA ≥ 36 weeks and birth weight ≥ 1800 g;
2. Clinical or paraclinical symptoms of asphyxia during delivery: Apgar score <5 at 5 minutes and/or a pH <7.00, and/or base deficit > -13 mmol/L, and/or lactate levels > 11 mmol /L in cord blood or in the first hour of life; and/or need for assisted ventilation (endotracheal or face mask) at 10 minutes of life;
3. Abnormal neurological examination according to Sarnat’s classification (Stage II or III);
4. Abnormal electrophysiology in standard EEG or amplitude-integrated EEG (low voltage, periodic and/or paroxysmal trace, convulsion);
5. HT started in the first six hours of life.

Due to missing data (46% of the data base population), criterion 4 concerning EEG was not taken into account. HT was qualified as i) optimal (OHT) if all other criteria were met, ii) late HT (LHT), if criteria 1 to 3 were met, but hypothermia was initiated more than 6 hours after birth, iii) non indicated HT (NIHT), if one or more of the 3 first clinical criteria was missing (either gestational age/birthweight, or asphyxia, or neurological examination), whatever the time to initiate hypothermia.

### Statistical Analysis

Several hypotheses were retained to deal with missing data. As "need for assisted ventilation at 10 minutes of life" (criteria 2) was not mentioned as such in the database, newborns requiring intubation and/or non-invasive ventilation with an Apgar score <10 at 10 min of life were considered to present this criterion. When data concerning the time to initiation of HT was missing (n = 16), the time between birth and admission to the NICU was used. Considering that this choice may underestimate this delay, a sensitivity analysis was performed: the alternative solution consisting in adding the mean time from admission to initiation of HT observed among babies with complete data was considered. The distribution of OHT, NIHT and LHT, and the factors associated with the practice of HT were reassessed according to this scenario.

As all French level III centers did not participate, main characteristics of participating and non-participating NICUs were compared.

Newborns were classed into three groups, OHT, LHT and NIHT, as defined above. We compared the organizational factors at birth, the newborns clinical and paraclinical characteristics, and the conditions in which HT was performed. The Chi^2^ test (or Fisher exact test if insufficient numbers) and Student's t test or an analysis of variance were used to analyze the qualitative and quantitative variables respectively. The threshold of type 1 risk was set at 5%. Statistical analysis was performed using STATA software (Stata / IC 10.0 for Windows).

## Results

Among the 68 NICU in France, 33 (49%) participated. These NICUs were more likely to be teaching Hospitals (75.8% versus 38.2%, p<10^−2^), but did not differ for the size of the maternity unit, the size of the neonatal unit or the type of intensive care (pediatric and neonatal intensive care, or exclusively neonatal intensive care) (data not shown).

For the whole study population, the main characteristics are presented in Table 1. The breakdown by severity of HIE into grade of I, II, III was 2%, 64% and 34%, respectively. Information regarding the intrapartum context associated with HIE was missing in 35% (n = 108) of cases. When details were recorded, the main complications were funicular pathologies (prolapse, circular loop) for 25% of cases, and dystocia during delivery for 19% of cases. A maternal infection was reported in 6% of cases. A life threatening event (at between 15 and 120 min of life) was reported for seven newborns.

**Table 1:** General characteristics of the whole study population.

Category	Subcategory	N	n (%) or mean +/– [SD]
Gestational age (weeks)		311	39.2 [1.5]
Birthweight (g)		311	3170 [547]
Male		311	163 (52.4)
Level of care in birth place	Home	311	4 (1.3)
	Level I	311	74 (23.8)
	Level II	311	125 (40.2)
	Level III	311	106 (34.1)
No identified obstetrical event		311	108 (34.7)
Apgar <5 at 5min		301	181 (60.1)
Severity of HIE	Mild	311	5 (1.6)
	Moderate	311	198 (63.7)
	Severe	311	108 (34.7)
EEG performed before hypothermia		301	168 (55.8)
Time to initiate hypothermia (hours)		308	5h41 [4h33]
Technique used to induce hypothermia	Criticool®	311	129 (41.5)
	Tecotherm®	311	90 (28.9)
	Artisanal*	311	51 (16.4)
	Méditherm®	311	24 (7.7)
	Blanketroll®	311	16 (5.1)
	Coolcap®	311	1 (0.3)
Abnormal MRI		284	155 (54.6)
In-hospital mortality		310	63 (20.3)
Abnormal neurological examination among survivors		238	69 (29.0)

: Switch off incubator or ice packs.

Our present study population is composed of 311 neonates, 202 (65%) in the OHT group, 68 (22%) in the LHT group, and 41 (13%) in the NIHT group (Figure 1). Table 2 shows the criteria involved in this classification. By definition, the clinical context in the group of newborns with NIHT is less severe than in the two other groups. The mean GA of babies in the NIHT group was one week lower (38.6w versus 39.3w), criteria suggesting asphyxia are lacking in more than one half of these babies, and HIE is considered as mild for 5 of them (12%). It should be noted that HT was initiated before 6 hours for only 71% of them. The groups of OHT and LHT are more comparable. About two thirds of neonates in these two subgroups presented severe HIE. EEG was more often performed in the group of OHT (55 versus 27%), and abnormal (97 versus 80%). Nearly 12 hours were needed before starting HT among newborns with LHT.

**Table 2:** Criteria indicating HT according to HT subgroups.

		OHT (n = 202)	LHT (n = 208)	NIHT (n = 41)	
Category	Sub category	n (%) or mean +/– [SD]	n (%) or mean +/– [SD]	n (%) or mean +/– [SD]	p
Gestational age (weeks)		39.3 [1.5]	39.3 [1.5]	38.6 [1.9]	*
Birthweight (g)		3201 [575]	3144 [475]	3073 [508]	ns
Asphyxia criteria	Apgar<5 at 5 min	129 (65.2)	43 (60.1)	9 (25.0)	***
	pH cord <7	98 (63.6)	24 (53.3)	7 (28.0)	**
	Lactate in cord >11mmol/L	89 (64.5)	24 (53.3)	4 (15.4)	***
	Base deficit in cord <–16	15 (46.9)	5 (62.5)	2 (40.0)	ns
	Ventilation	146 (72.3)	54 (79.4)	16 (39)	***
	Intubation	172 (85.1)	53 (77.9)	24 (58.5)	***
	Chest Compression	85 (42.1)	23 (33.8)	11 (26.8)	ns
	Adrenalin	58 (28.7)	12 (17.6)	11 (26.8)	ns
Severity of HIE	Mild	0	0	5 (12.2)	***
	Moderate	131 (64.9)	44 (64.7)	23 (56.1)	Ns
	Severe	71 (35.1)	24 (35.32)	13 (31.7)	ns
EEG performed before hypothermia		111 (54.9)	18 (26.5)	14 (34.2)	***
Abnormal EEG		84 (96.5)	39 (79.6)	23 (88.5)	**
Age at start of hypothermia (h)		3h35 [1.36]	11h54 [3.21]	5h41 [1.10]	***
Hypothermia started before 6h		202 (100)	0	27 (71.1)	***

OHT: optimal hypothermia treatment, LHT: late hypothermia treatment, NIHT: non indicated hypothermia treatment, *** p<10^−3^, **10^−3^≤p<10^−2^, *10^−2^≤p<0.05,

ns : not significant.

Table 3 shows different factors (organization of care, temperature at admission, and obstetrical circumstances) associated with the different groups of HT. Inborn birth tended to be more common in the OHT group. The size of the obstetric unit and the frequency of birth outside office hours (18h-8H) were similar between the three groups. The late initiation of HT in the LHT group was the result of a long delay between admission and treatment (nearly 8 hours compared to less than 1 hour in the OHT group, p<10-3). The mean temperature at admission to NICU was lower in the group of OHT compared with the two others (34.9 versus 35.74 and 35.3). An acute obstetrical event was more likely to be identified among newborns with LHT (46%) compared to OHT (34%) and NIHT (22%). Nevertheless, apart from uterine rupture, no other circumstance of delivery differed significantly between the three groups. None of HIE in the OHT and LHT groups were post-mature births, while 17% of HIE were concerned.

**Table 3:** Organization of care and birth circumstances according to HT subgroups.

		OHT (n = 202)	LHT (n = 208)	NIHT (n = 41)	
Category	Sub category	n (%) or mean +/– [SD]	n (%) or mean +/– [SD]	n (%) or mean +/– [SD]	p
Newborn characteristics	inborn	62 (30.7)	15 (22.1)	8 (19.5)	ns
	male	102 (50.5)	37 (54.4)	24 (58.5)	ns
	Temperature at admission to NICU (°C)	34.9 [1.4]	35.4 [1.4]	35.3 [1.4]	ns
Size of maternity	Less than1000 births/year	23 (11.4)	3 (4.4)	4 (9.8)	ns
	1000 – 2000	0	0	1 (2.4)	ns
	2000 – 3000	79 (39.1)	26 (38.2)	17 (41.5)	ns
	3000 – 4000	76 (37.6)	28 (41.2)	14 (34.1)	ns
	Over 4000	24 (11.8)	11 (16.2)	5 (12.2)	ns
Delay	Age at start of hypothermia (h)	3h35 [1.36]	11h54 [3.21]	5h41 [1.1]	***
	Time between birth and admission (h)	2h54 [2.28]	3h36 [2.2]	3h17 [2.08]	***
	Time between admission and start of HT	0h44 [2.43]	7h56 [5.28]	3h09 [3.5]	***
Obstetrical Circumstances	Funicular cause	34 (16.8)	11 (16.2)	6 (14.6)	ns
	Dystocia	25 (12.4)	7 (10.3)	6 (14.6)	ns
	Retro-placental hematoma	24 (11.9)	7 (10.3)	3 (7.3)	ns
	Uterine rupture	22 (10.9)	2 (2.9)	1 (2.4)	*
	Infection	8 (4.0)	2 (2.9)	3 (7.3)	ns
	Velamentous insertion of the cord	7 (3.5)	1 (1.5)	2 (4.9)	ns
	Feto-maternal hemorrhage	5 (2.5)	1 (1.5)	2 (4.9)	ns
	Post-maturity	0	0	7 (17.1)	***
	No causal circumstances found or reported	68 (33.7)	31 (45.6)	9 (22)	*

OHT: optimal hypothermia treatment, LHT: late hypothermia treatment, NIHT: non indicated hypothermia treatment, *** p<10^−3^, **10^−3^≤p<10^−2^, *10^−2^≤p<0.05,

ns : not significant,

There was no difference in the method used to achieve HT (ice packs, switching off the incubator) and in the duration of HT between the groups (Table 4). There was a gradation in the rates of adverse events and complications from the NIHT group to the LHT group and the OHT group, although this increase did not reach significance for adverse events. About one in five infants suffered adverse events during HT. One half of newborns in the OHT group presented at least one complication, the most frequent being liver failure or cytolysis (28.7%), renal insufficiency (24.8%), shock (24.3%), and pulmonary hypertension (11.4%). Three in ten newborns with NIHT suffered complication(s). No significant difference was observed between the types of complications linked to HI in each subgroup.

**Table 4:** Characteristics of HT, adverse events, complications and short-term outcomes according to the different HT subgroups.

		OHT (n = 202)	LHT (n = 208)	NIHT (n = 41)	
Category	Sub category	n (%)	n (%)	n (%)	p
Procedural for HT	Hypothermia continued for 72h	180 (29.1)	58 (85.3)	36 (87.8)	ns
	No special hypothermia equipment ^+^	32 (15.8)	12 (17.6)	7 (17.1)	ns
Adverse events associated with HT	At least one adverse event	58 (29)	16 (23.5)	7 (17.1)	ns
	Thrombopenia	16 (8)	6 (8.8)	4 (9.8)	ns
	Hypomagnesemia	9 (4.5)	5 (7.4)	0	ns
	Excessive cooling	12 (6)	5 (7.4)	0	ns
	Cutaneous lesions	2 (1.0)	0	0	ns
	Hemorrhage	10 (5.0)	3 (4.4)	0	ns
Complications linked to HI	At least one complication	105 (52.0)	36 (38.2)	12 (29.3)	**
	Liver failure/cytolysis	58 (28.7)	13 (19.1)	6 (14.6)	ns
	Shock	49 (24.3)	17 (25.0)	5 (12.2)	ns
	Hypertension	15 (7.4)	2 (2.9)	1 (2.4)	ns
	Renal insufficiency	50 (24.8)	10 (14.7)	5 (12.2)	ns
	Pulmonary hypertension	23 (11.4)	7 (10.7)	3 (10.3)	ns
	Hypotension	7 (3.5)	2 (2.9)	1 (2.4)	ns
	Glucose intolerance	4 (2.0)	1 (1.5)	3 (7.3)	ns
	Ileus	1 (0.5)	0	0	ns
Abnormal RMI	All lesions	110 (59.5)	27 (43.5)	18 (46.6)	ns
	Basal Ganglia and/or cortical or subcortical	80 (43.2)	19 (30.6)	16 (43.2)	ns
	White matter lesions apart from hemorrhagic petechial	43 (23.2)	12 (19.4)	9 (24.3)	ns
	Isolated hemorrhagic petechial lesions	18 (9.7)	4 (6.4)	0	ns
	Brainstem or cerebellar lesion	11 (5.9)	3 (4.8)	1 (2.7)	ns
In-hospital mortality	All causes	45 (22.3)	10 (14.9)	8 (19.5)	ns
	Neurological cause without limitation of care	6 (3.0)	0	1 (2.4)	ns
	Neurological cause with limitation of care	36 (17.8)	9 (13.4)	7 (17.1)	ns
	Other cause	3 (1.5)	1 (1.5)	0	ns
Neurological examination for survivors	Normal at discharge from NICU	104 (68.9)	42 (75)	23 (74.2)	ns
	Moderate at discharge from NICU	43 (28.5)	13 (23.2)	7 (22.6)	ns
	Severe at discharge from NICU	4 (2.6)	1 (1.8)	1 (3.2)	ns

OHT: optimal hypothermia treatment, LHT: late hypothermia treatment, NIHT: non indicated hypothermia treatment, +Switch off incubator or ice packs, *** p<10^−3^,

10^−3^≤p<10^−2^, *10^−2^≤p<0.05, ns : not significant.

The rates of death or abnormal neurological examination at discharge were similar between the three groups. In contrast, nearly 60% of newborns in the OHT group had an MRI showing abnormalities compared to 44% and 49% in the LHT and NIHT groups respectively. No difference in the type of abnormalities was found.

Finally, we performed a sensitivity analysis consisting in adding the mean time between admission and initiation of HT observed among babies with complete data, when time to initiate HT was unknown. Among the 16 cases concerned, 4 were initially classified as NIHT, 1 as LHT and 11 as OHT. Under the hypothesis tested as part of the sensitivity analysis, the first 5 children remained in the same group. In contrast, time to initiate hypothermia became greater than 6 hours for 4 of the remaining 11 children, and these newborns were reclassified in the LHT group. The corresponding rates of OHT and LHT were respectively 64 (n = 198) and 23% (n = 72). This new distribution did not affect our previous findings about the factors associated with the different HT subgroups.

## Discussion

This study shows that for 35% of newborns with HIE and treated with HT the conduct of the treatment is not consistent with the guidelines published by the French Society of Neonatology. The main reason for non-compliance (62%) is a delay in the start of treatment, beyond the 6 hours recommended. The second reason is the lack of adherence to the clinical indications for HT (38%). Apart from late transfer to the NICU, the main characteristics of newborns in the SOHT group are reduced severity of HIE and less frequent need for resuscitation in the delivery room. Finally, the complications generally associated with HT are significantly less frequent in the groups LHT and NIHT.

In our study, HT was started at or over 6 hours of life for 22%, and at or over 8 hours of life for 18% of all neonates. This rate is higher than that in two recent studies evaluating the practice of HT in the UK and in Belgium and The Netherlands, estimated at about 5%, and at 1% of newborns between 8 and 12 hours of life [9], [26].However, the study design, inclusion criteria and/or the characteristics of participating centers (centers with a high level of awareness to HT due to their participation in large randomized studies), might explain the disparities with our study which reflects the daily practice of French NICUs.

Regarding adverse events associated with HT and complications linked to HI, our findings are similar to previous studies [9], [26]. It is difficult to distinguish those associated with the natural history of HIE and those particularly related to HT. In our opinion, the most severe side effects attributable to HT are cysteatonecrose and persistent pulmonary hypertension. In our sample, these adverse events were rare, respectively 2 and 5 cases. Concerning the NIHT, the adverse events were comparable to those of other groups. Among the 5 cases of mild HIE treated with HT, no adverse events were observed and only one newborn presented several complications.

Most treatment guidelines recommend a delay of less than 6 hours between birth and the start of HT treatment. The effectiveness of late treatment, initiated between 6 and 24 hours is currently under study, but in the absence of known results, it cannot be recommended at present [27]. The ways to reduce the delay before starting HT remain unclear, particularly in the context of an unpredictable acute perinatal pathology. Reducing the time of transfer by ambulance to the NICU is probably unfeasible in France. Nevertheless, doctors in charge of the ambulance services should be reminded of the urgency to be given to calls for neonatal neurological distress, to ensure rapid transfer of the newborn to a level III NICU. The initiation of HT during transport prior to arriving at the NICU is proposed by some teams and several studies have tested this solution. This strategy sometimes obtains the target temperature of 33.5 ° C, either by passive or active HT [28]. There is however a risk of excessively lowering HT to below 33.5 ° C, which is limited by means of the development of mobile devices. The drawback of this solution is to compromise the interpretation of the first EEG [29], while in most randomized trials about cooling, an electrophysiological examination before hypothermia is recommended so as confirm the indication for HT, increasing thus avoiding the risk of over-treatment.

Failure to recognize the indications for HT is the second reason for non-adherence to the guidelines. In our study, there were 41 newborns (13.2%) for whom no clinical or biological signs of perinatal asphyxia were recorded. Of these, the GA at birth was <36 weeks for four, four others did not show all the neurological signs of HIE, and for two newborns two or more of these three criteria were missing. Some authors suggest that the indication for HT should be extended to moderately premature infants (34–36 weeks GA), or to infants with less severe HIE [30]. A feasibility study of brain HT for preterms between 32 and 36 weeks is ongoing [31]. Currently, the level of evidence does not justify an extension of the indication. We observed that 46% of newborns had no electrophysiological examination prior to HT. Difficulties in performing and obtaining an interpretation of a standard EEG, especially during the night may partly explain this result. Greater dissemination of amplitude integrated EEG, which is simpler to implement and interpret, should make it easier to obtain an electrophysiological assessment before treatment [32]. Furthermore, the sensitivity and specificity in assessing the prognosis is between 70 and 100% depending on the study [33], [34].

For 35% of our population, no perinatal circumstances that could explain the intrapartum asphyxia are mentioned in the database. This has already been observed in another French study for 19% of newborns with stage II or III encephalopathy [5]. In the randomized trial of Shankaran *et al*, a complication during delivery was only noted in 68 cases among the 102 newborns treated with HT [35]. This raises the question of the anoxic-ischemic character of encephalopathy for some cases in our series. Moreover, the designation “HIE” is debated in the literature, some authors preferring to use the term “neonatal encephalopathy” in view of the difficulty in proving an anoxic-ischemic cause of neonatal neurological distress.

A major limitation of our study is the declarative nature of the database. It is likely that the population of newborns treated for HIE is selected and not completely representative, but the aspects influencing the selection and thus how they influenced our results are undocumented. Therefore the SFN database does not necessarily reflect the general practice of HT in France, as the registration of all cases is still not achieved. To maximize the number of reported cases, we contacted all the centers in December 2011. For the 26 most recently reported cases, most (n  =  20) were classified in the OHT group suggesting some improvement in practice over time. Our management of missing data may have generated a classification bias. Concerning the time to initiate HT, we have checked through the sensitivity analysis to ensure that our strategy, which potentially underestimated this delay, was not likely to change our findings. As electrophysiology results were not available for 46% of cases, this criterion could not be used for this study while it is crucial for determining the severity of HIE and thus in classing the newborns.

## Conclusions

This is the first multicenter French study to look at the practice of HT nationally. Although the results should be treated with caution since our database is only declarative, it reveals non-compliance with the guidelines in 35% of cases. These correspond to excess treatments. Our results attest to the difficulty of implementing the guidelines in clinical practice. This initial study should prompt a better organization of perinatal care, in particular to reduce the transportation time of newborns with HIE, as well as the diffusion of mobile equipment allowing the early initiation of HT in safe conditions. We also suggest that more systematic use of amplitude EEG examinations in neonatal units could improve the rate of electrophysiological assessment before HT treatment. Finally this audit of daily practice might prompt the widespread use of this recent neuro-protective strategy, and improve the awareness of HT among neonatologists.
